# ABCB1 polymorphism predicts the toxicity and clinical outcome of lung cancer patients with taxane‐based chemotherapy

**DOI:** 10.1111/1759-7714.13184

**Published:** 2019-09-30

**Authors:** Jia Zhong, Zihan Guo, Liping Fan, Xinghui Zhao, Bingqing Zhao, Zhigang Cao, Linlin Cheng, Yuanyuan Shi, Xiaoting Li, Yanhua Zhang, Tongtong An, Meina Wu, Yuyan Wang, Minglei Zhuo, Jianjie Li, Xue Yang, Hanxiao Chen, Bo Jia, Jun Zhao

**Affiliations:** ^1^ Key Laboratory of Carcinogenesis and Translational Research (Ministry of Education), Department of Thoracic Medical Oncology‐I Peking University Cancer Hospital & Institute Beijing China; ^2^ Key Laboratory of Carcinogenesis and Translational Research (Ministry of Education), Department of Pharmacy Peking University Cancer Hospital & Institute Beijing China; ^3^ Department of Medical Oncology Mancheng People's Hospital Baoding China; ^4^ Deportment of Medical Oncology Dong'e People's Hospital Liaocheng China; ^5^ Key Laboratory of Carcinogenesis and Translational Research (Ministry of Education), Department of Radiology Peking University Cancer Hospital & Institute Beijing China

**Keywords:** ABCB1, chemotherapy, lung cancer, pharmacokinetic

## Abstract

**Background:**

Taxane‐based chemotherapy is widely used in lung cancer. ABCB1 have a role in the prediction of treatment response and toxicity of chemotherapy in solid tumors. In this retrospective study, we investigated ABCB1 polymorphism on response and toxicity in taxane‐based chemotherapy in lung cancer patients.

**Methods:**

A total of 122 lung cancer patients who received taxane‐based chemotherapy were included in this study. Fluorescence in situ hybridization (FISH) was used for ABCB1 polymorphism detection. Turbidimetric inhibition immunoassay was used for pharmacokinetic analysis. Statistical analysis was performed using SPSS 20.0.

**Results:**

The frequency of the ABCB1 2677 site TT/TG/GG genotype was 32.8%, 43.4% and 23.8%, respectively and the frequency of the 3435 sites the TT/TC/CC genotype was 13.9%, 44.3% and 41.8%, respectively. The occurrence of neurotoxicity was higher in patients who had ABCB1 3435 site mutation (TT 88.2%, TC 22.2%, CC 21.6% *P* = 0.004). There was no significant difference between ABCB1 genotypes with regard to other chemotherapy‐induced toxicity. For non‐small cell lung cancer (NSCLC) patients, those harboring ABCB1 2677 and 3435 site wild‐type patients had longer median progression‐free survival (PFS) in the paclitaxel subgroup (3435 site: TT 3.87 vs. TC 9.50 vs. CC 14.13 months; *P* < 0.001; 2677 site: TT 4.37 vs. TG 9.73 vs. GG 12.1 months; *P* = 0.013). The area under the concentration‐time curve (AUC) of 20 patients treated with docetaxel increased for ABCB1 mutation subgroups.

**Conclusion:**

ABCB1 mutation is associated with higher neurotoxicity of taxane‐based chemotherapy. It also predicts shorter PFS for NSCLC in paclitaxel‐based treatment.

## Introduction

Taxanes are anticancer drugs with a wide range of therapeutic application and are commonly used to treat solid tumors. However, challenges still exist because of the considerable variability in efficacy and toxicity among different individuals. Previous studies have indicated that such interpatient variability is linked to polymorphisms in genes encoding drug transporters and drug‐metabolizing enzymes.^1^

One of the crucial proteins involved in taxane metabolism and distribution is the ATP‐binding cassette transporter B1 (ABCB1, P‐glycoprotein, MDR1). ABCB1 is a transmembrane protein that can act as an ATP‐driven drug export pump for chemotherapeutic agents, such as taxanes.^2^ Moreover, ABCB1 has been reported to be an important factor in the survival and proliferation of epithelial cells and malignant cells during tumorigenesis.^3^ There are more than 50 single nucleotide polymorphisms (SNPs) reported in the ABCB1 gene, and at least two of these variants (2677G > T/A and 3435C > T) have been associated with altering the expression of P‐glycoprotein (P‐gp).^4^, ^5^ P‐glycoprotein functions as an energy‐dependent drug efflux pump. The taxanes can be extruded by P‐glycoprotein through cell membranes and high expression of P‐glycoprotein on tumor cells increases the efflux of chemotherapeutic drugs from tumor cells, thus leading to a resistant phenotype.^6^

Previous studies have reported that response to paclitaxel in ovarian cancer patients together with paclitaxel‐induced neutropenia and neuropathy were related to biallelic polymorphism at the 2677 and 3435 loci of ABCB1.^6^, ^7^ Another study showed that ABCB1 2677 and 3435 variants in advanced breast cancer patients were associated with disease control rates and overall survival.^8^ In gastric cancer ABCB1 polymorphism at 3435 sites was also related to clinical outcomes after paclitaxel‐contained chemotherapy.^9^

However, no previous studies have indicated these two variants of ABCB1 in association with response or toxicity for taxane‐based therapy in lung cancer. In this study, we evaluated the frequency of ABCB1 polymorphisms in lung cancer patients and studied whether the ABCB1 polymorphisms were associated with clinical outcomes or toxicity of taxane‐based chemotherapy.

## Methods

### Patients

This retrospective study enrolled a total of 122 consecutive Chinese patients. The inclusion criteria were male or female patients aged 18 years or older, at least one lesion that could be accurately measured according to the Response Evaluation Criteria in Solid Tumors (RECIST 1.1), and patients who received taxane‐based (paclitaxel, docetaxel, paclitaxel‐albumin) chemotherapy. Cytological or histological confirmation of non‐small cell lung cancer (NSCLC); only patients with locally advanced (phase IIIb but could not receive the local treatment) or metastatic when receiving taxane‐based chemotherapy were included in the survival analysis. These patients were diagnosed and treated at Peking University Cancer Hospital between August 2015 and June 2017. Among the 122 patients, 20 cases who received docetaxel treatment accepted additional ancillary pharmacokinetic analysis.

This study was reviewed and approved by the Institutional Ethics Committee at Peking University Cancer Hospital. All patients had provided written informed consent before specimens were collected.

The RECIST version 1.1 was used for response evaluation and patients were assessed for response evaluation after every two cycles of treatment. Toxicity was defined by National Cancer Institute Common Terminology Criteria for Adverse Events (CTCAE), version 4.02. Toxicity assessments were performed each cycle, which included the recording of symptoms and vital signs, hematologic and biochemical laboratory testing. PFS was calculated from the beginning of treatment to the date of tumor progression or death. Patients without a known date of death or progression were censored at the time of the last follow‐up.

### ABCB1 detection

A total of 4 mL blood from each patient was provided for the study. We collected blood samples in EDTA anticoagulation tubes and these were stored at −80°C until analysis. Genomic DNA was isolated from blood samples using the nucleic acid purification kit (Sino‐Era: Gene Tech, China) and stored at −20°C until use. Genotype screening was performed using the universal sequencing kit (Sino‐Era: Gene Tech, China).

### Pharmacokinetic analysis

A pharmacokinetic analysis of 20 patients who receive docetaxel treatment was carried out. During the first cycle of docetaxel chemotherapy, 2 mL venous blood samples were collected in tubes with dipotassium ethylenediaminetetraacetic acid (EDTA) as an anticoagulant within 10 minutes before the end of infusion (sample 1) and 30–60 minutes after the end of infusion (sample 2). Docetaxel in plasma was immediately quantified by MyDocetaxel kit (Shanghai Fosun Long March Medical Science Co. Ltd, China) according to the protocol described by the manufacturer. According to the results, pharmacokinetic parameter, in other word area under the concentration‐time curve (AUC), was calculated by using the Drug Exposure Calculator TM (Version 1.0; Thermo Fisher Scientific, USA).

### Statistical analysis

Statistical analyses were performed using SPSS 20.0 software (SPSS Inc., Chicago, IL, USA). The relationships between the ABCB1 and relevant factors such as age, gender, smoking status and pathology were examined using chi‐square tests in which *P* < 0.05 represented a bilateral significant difference. The relationships between the ABCB1 and PFS were analyzed using Kaplan‐Meier survival curves. The prognostic factors for PFS after taxane treatment were analyzed using Cox regression analysis. We used SHEsis, a software platform for analyses of linkage disequilibrium (http://analysis.bio-x.cn/myAnalysis.php).

## Results

### Patient characteristics and frequencies of genotypes

There were a total of 122 patients who received taxane treatment for ABCB1 detection. The median age of the patients was 61 years (range 34–82) and there were 105 men (86.1%) and 17 women (13.9%). A total of 47 (38.5%) patients were adenocarcinoma, 56 (45.9%) patients were squamous cell lung cancer, nine (7.4%) were small cell lung cancer and 10 (8.2%) were other lung cancer subtypes. At initial diagnosis, 75 (61.5%) patients had stage IV disease and 47 (38.5%) patients had stage I–III disease. All patients included in this study received palliative chemotherapy. adjuvant chemotherapy was not included for effect evaluation. A total of 53 (43.4%) patients received paclitaxel, 65 (52.5%) docetaxel, and five (4.1%) paclitaxel‐albumin. A total of 74 (60.7%) patients received platinum‐based chemotherapy (Table 1). Amongst these, 68 (55.7%) were first‐line and 54 (44.3%) late‐line. A total of 60 (49.2%) had a combined regimen and 62 (50.8%) a single agent. The relationship between genotypes and response are listed in Table 2.

**Table 1:** Patient characteristics and genetic polymorphisms

	Total	2677SNP			3435SNP			*P*‐value 2766
Characteristics	*n* = 122 (%)	TT/TA/AA (*n* = 29)	GT/GA (*n* = 53)	GG (*n* = 40)	TT (*n* = 17)	TC (*n* = 54)	CC (*n* = 51)	/ *P*‐value 3435
Age								
Median (range)	61 (34–82)	60 (41–76)	61 (34–82)	60 (48–82)	61 (45–76)	60.5 (34–82)	60 (41–76)	0.352/0.789†
Gender								
Male	105 (86.1%)	23 (79.3%)	46 (86.8%)	36 (90%)	13 (76.5%)	47 (87.0%)	45 (88.2%)	0.440/0.461‡
Female	17 (13.9%)	6 (20.7%)	7 (13.2%)	4 (10%)	4 (23.5%)	7 (13.0%)	6 (11.8%)	
Pathology								
Adenocarcinoma	47 (38.5%)	7 (24.1%)	23 (43.4%)	17 (42.5%)	4 (23.5%)	23 (42.6%)	20 (39.2%)	0.424/0.188‡
Squamous cell carcinoma	56 (45.9%)	15 (51.7%)	23 (43.4%)	18 (45.0%)	8 (47.1%)	23 (42.6%)	25 (49.0%)	
Small cell	9 (7.4%)	4 (13.8%)	4 (7.5%)	1 (2.5%)	4 (23.5%)	3 (5.6%)	2 (3.9%)	
Others	10 (8.2%)	3 (10.3%)	3 (5.7%)	4 (10.0%)	1 (5.9%)	5 (9.3%)	4 (7.8%)	
Stage at diagnosis								
I–II	2 (1.6%)	0 (0.0%)	0 (0.0%)	2 (5.0%)	0 (0.0%)	0 (0.0%)	2 (3.9%)	0.247/0.425‡
III	45 (36.9%)	11 (37.9%)	23 (42.5%)	11 (27.5%)	7 (41.2%)	23 (41.5%)	15 (29.4%)	
IV	75 (61.5%)	18 (62.1%)	30 (57.5%)	27 (67.5%)	10 (58.8%)	31 (58.5%)	34 (66.7%)	
Taxane								
Paclitaxel	53 (43.4%)	9 (31.0%)	28 (52.8%)	16 (40.0%)	5 (29.4)	26 (48.1%)	22 (43.1%)	0.272/0.564‡
Paclitaxel‐albumin	5 (4.1%)	1 (3.4%)	3 (5.7%)	1 (2.5%)	1 (5.9%)	3 (5.6%)	1 (2.0%)	
Docetaxel	65 (52.5%)	19 (65.5%)	22 (41.5%)	23 (57.5%)	11 (64.7%)	25 (46.3%)	28 (54.9%)	
Smoking								
Smoker or ever smoker	91 (74.6%)	22 (75.9%)	42 (79.2%)	26 (65.0%)	35 (68.6%)	42 (77.8%)	14 (82.4%)	0.366/0.561‡
Non‐smoker	31 (25.4%)	7 (24.1%)	10 (18.9%)	14 (35.0%)	3 (17.6%)	12 (22.2%)	16 (31.4%)	

† *t*‐test.

‡ Pearson chi‐square.

**Table 2:** The relationship between genotypes and response

		2677 SNP			3435 SNP			
Best response	Total *n* = 122(%)	TT/TA/AA (*n* = 29)	GT/GA (*n* = 53)	GG (*n* = 40)	TT (*n* = 17)	TC (*n* = 54)	CC (*n* = 51)	*P*‐value 2766/*P*‐value 3435
PR	25 (20.5)	5 (20.0)	16 (64.0)	4 (16.0)	3 (12.0)	15 (60.0)	7 (28.0)	0.000/0.001
SD	51 (41.8)	12 (23.5)	20 (39.2)	19 (37.3)	6 (11.8)	21 (41.2)	24 (47.1)	0.187/0.000
PD	22 (18.0)	7 (31.8)	5 (22.7)	10 (45.5)	4 (18.2)	9 (40.9)	9 (40.9)	0.274/0.182

PR, partial reponse; SD, stable disease; PD, progression disease.

**Figure 1:**
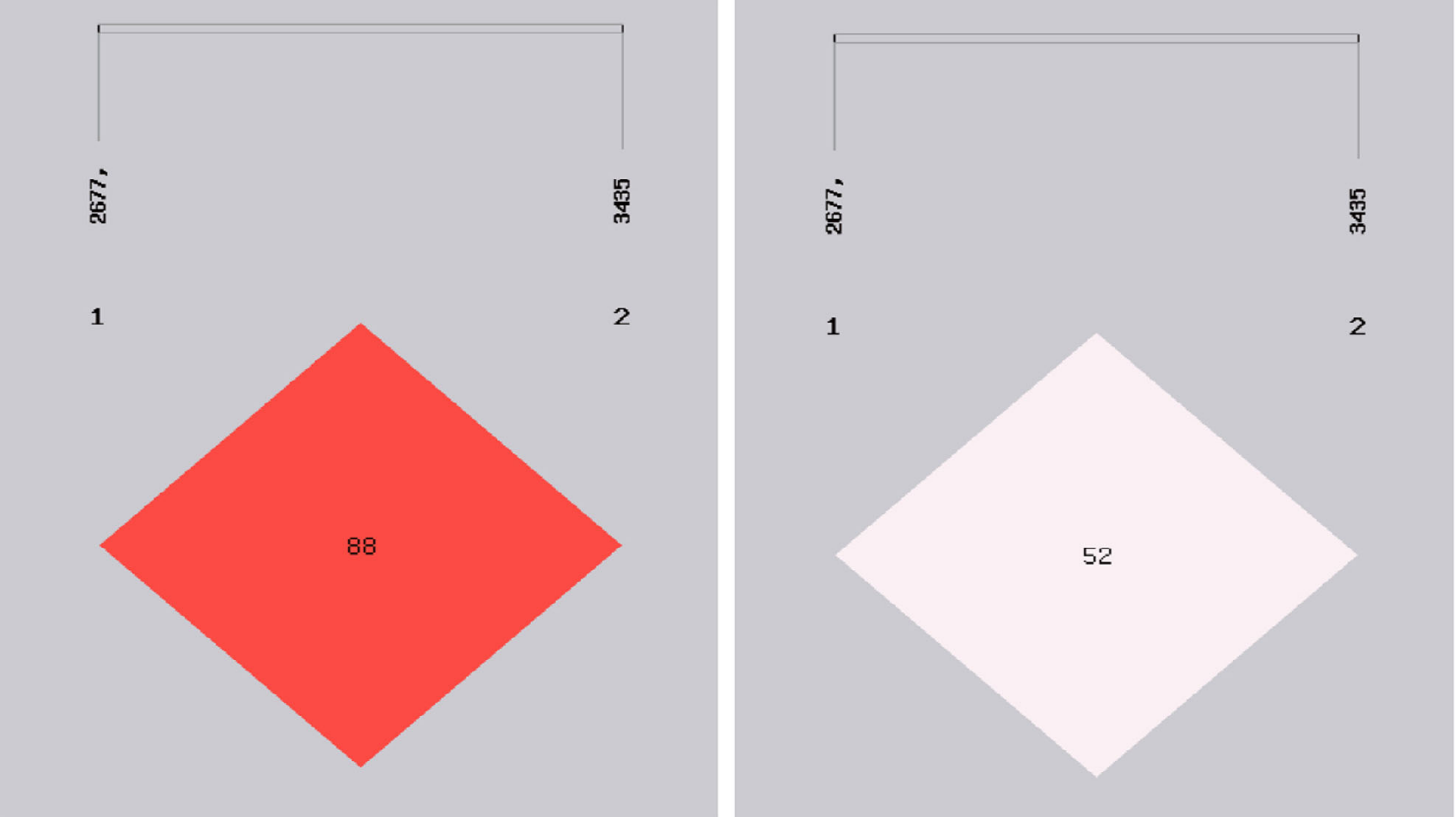
Linkage disequilibrium test of SNPs of ABCB1 2677 G > T/A locus and 3435 C > T locus (r2 = 0.528, D' = 0.884, D' is the value of D prime between the two loci. r2 is the correlation coefficient between the two loci).

For the ABCB1 2677 G > T/A locus, the percentage of patients with the TT/TG/GG genotype was 32.8%, 43.4% and 23.8%, respectively. For the ABCB1 3435 C > T locus, the percentage of patients with the TT/TC/CC genotype was 13.9%, 44.3% and 41.8%. The distribution frequency of ABCB1 2677 G > T/A (chi‐square = 1.877; *P* = 0.204) and 3435 C > T (chi‐square = 0.197; *P* = 0.709) genotype conformed to Hardy‐Weinberg equilibrium. Using SHEsis analysis, we observed high linkage disequilibrium (LD) of 2677 G > T/A locus and 3435 C > T locus. r^2^ (squared allele‐frequency correlations) equal to 0.528 and D' (standardized disequilibrium coefficients) equal to 0.884, were both higher than the cutoff value (r^2^ > 0.33, D' > 0.7 indicated high LD) (Fig 1).

No association was found between any ABCB1 alleles and age, sex, stage, pathology, smoking and EGFR mutation status. The genotype and allele frequencies for each SNP are reported in Table 3.

**Table 3:** Genetic polymorphism frequency

Variant	Classifications	Frequency
ABCB1 2677 G > T/A‡	TT，TA and AA	40 (32.8%)
	GT and GA	53 (43.4%)
	GG	29 (23.8%)
ABCB1 3435 C>T‡	TT	17 (13.9%)
	TC	54 (44.3%)
	CC	51 (41.8%)
ABCB1 2677 Allele§	T/A	133 (54.5%)
	G	111 (45.5%)
ABCB1 3435 Allele§	T	88 (36.1%)
	C	156 (63.9%)
Diplotype‡	2677G/3435C‐2677G/3435C	37 (30.3%)
	2677T/A/3435T‐2677 T/A/3435T	15 (12.3%)
	2677†/3435†‐2677†/3435†	70 (57.4%)

† Any combination of alleles that is not mutually exclusive with another diplotype consisting of the 2677 and 3435 SNPs.

‡ Number represents number of patients (percentage).

§ Number represents number of alleles (percentage).

### ABCB1 polymorphisms and chemotherapy‐induced toxicities

We evaluated the association between ABCB1 polymorphisms and toxicities from all 122 lung cancer patients. No association was found between any ABCB1 alleles and constipation, diarrhea, leucopenia, neutropenia, thrombocytopenia, anemia, nausea, vomiting, myalgia, neuritis, and liver dysfunction (Table 4).

**Table 4:** ABCB1 polymorphisms and taxane‐induced toxicities

	2677 G > T				3435 C > T			
AE	TT (*n* = 40)	TG (*n* = 53)	GG (*n* = 29)	*P*‐value*	TT (*n* = 17)	TC (*n* = 54)	CC (*n* = 51)	*P*‐value*
Constipation	0 (0%)	2 (3.8%)	2 (6.9%)	0.497	2 (11.8%)	2 (3.7%)	0 (0%)	0.714
Diarrhea	2 (5.0%)	3 (5.7%)	0 (0%)	0.270	1 (5.9%)	4 (7.4%)	0 (0%)	0.244
Leucopenia	20 (50.0%)	31 (58.5%)	12 (41.3%)	0.323	10 (58.8%)	28 (51.9%)	25 (49.0%)	0.782
Neutropenia	19 (47.5%)	31 (58.5%)	12 (41.3%)	0.293	9 (52.9%)	29 (53.7%)	24 (47.1%)	0.779
Thrombocytopenia	2 (5.0%)	5 (9.4%)	3 (10.3%)	0.905	1 (5.9%)	5 (9.3%)	4 (7.8%)	0.900
Anemia	2 (5.0%)	6 (11.3%)	2 (6.9%)	0.523	2 (11.8%)	6 (11.1%)	2 (3.9%)	0.344
Nausea	15 (37.5%)	23 (43.4%)	10 (34.5%)	0.864	9 (52.9%)	21 (38.9%)	18 (35.3%)	0.598
Vomit	5 (12,5%)	7 (13.2%)	3 (10.3%)	0.731	2 (11.8%)	8 (14.8%)	5 (9.8%)	0.874
Myalgia	4 (10%)	5 (9.4%)	2 (6.9%)	0.675	1 (5.9%)	4 (7.4%)	6 (11.8%)	0.562
Neuritis	19 (47.5%)	14 (26.4%)	5 (17.2%)	0.073	15 (88.2%)	12 (22.2%)	11 (21.6%)	0.004
Liver dysfunction	6 (15.0%)	10 (18.9%)	4 (13.8%)	0.903	3 (17.6%)	9 (16.7%)	8 (15.7%)	0.962

* *P*‐value was calculated by chi‐squared trend analysis.

However, we found that patients carrying ABCB1 3435 C > T SNP were more likely to experience peripheral neuropathy (TT 88.2%, TC 22.2%, CC 21.6% *P* = 0.004). Although the 2677GG and 2677GT were statistically different from one another when individuals carrying the 2677 TT genotype were compared to individuals carrying 2677GG and 2677GT (TT 47.5%, TG 26.4%, GG 17.2%), the difference was not statistically significant.

To further analyze the association between neuritis and ABCB1 polymorphisms, we subdivided toxicities into none (CTCAE grade 0), mild (CTCAE grade 1–2) and severe (CTCAE grade 3–4). Generally, most neuritis was mild. The results were similar to the previous two classification analysis (Table 3) for genotype analysis. It was notable that the same patient carrying diplotype 2677T/A/3435T‐2677 T/A/3435T was more likely to have neuritis when compared to diplotype 2677G/3435C‐2677G/3435C and all the others population (*P* = 0.016) (Table 5).

**Table 5:** Association between ABCB1 polymorphisms and chemotherapy‐induced neuritis

	None (0)	Mild (1–2)	Severe (3–4)
2677 Genotype			
GG (*n* = 29)	15	5	0
TG (*n* = 53)	39	12	2
TT (*n* = 40)	21	18	1
*P*‐value	*P* = 0.099*		
3435 Genotype			
CC (*n* = 51)	36	11	0
TC (*n* = 54)	42	10	2
TT (*n* = 17)	6	14	1
*P*‐value	*P* = 0.008*		
Diplotype			
2677G/3435C‐2677G/3435C	27	9	1
Others	52	16	2
2677T/A/3435T‐2677 T/A/3435T	5	10	0
	*P* = 0.016*		

* *P*‐value was calculated by chi‐squared trend analysis.

### ABCB1 polymorphisms and treatment outcomes

Of the 122 patients enrolled, 104 were fully eligible for response evaluation. Eighty‐two patients had disease progression or had died at the last time of follow‐up. The median follow‐up time was 10.2 months (range, 0.6–118.6 months). We did not find a difference in either disease control rate or response rate between the different genotypes, or haplotypes for the non‐small cell lung cancer patients treated with docetaxel and paclitaxel‐albumin.

**Figure 2:**
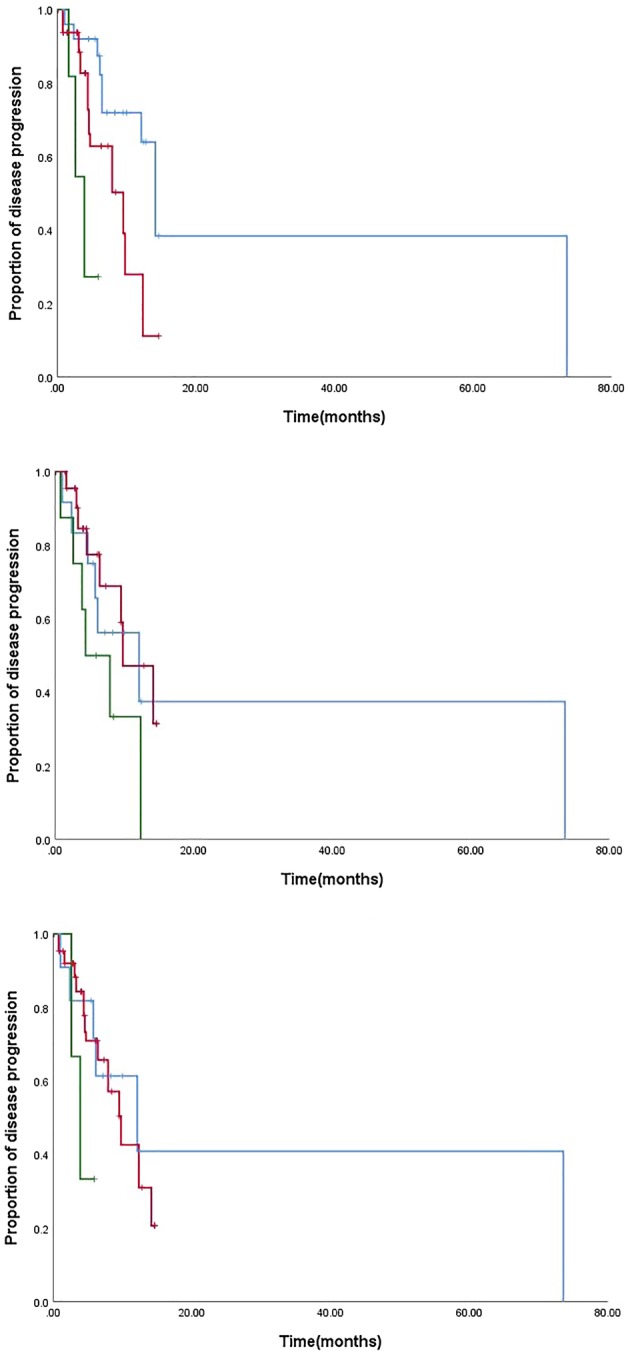
Correlation with ABCB1 and PFS of chemotherapy in paclitaxel treatment group. (**a**) PFS analysis in ABCB1(3435T>C) locus (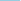) CC, (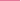) TC, and (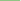) TT; (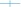) CC, (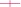) TC, and (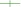) TT; (**b**) PFS analysis in ABCB1 2677 G>T/A locus (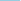) GG, (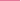) TG, and (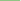) TT; (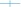) GG, (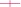) TG, and (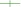) TT; (**c**) PFS analysis in ABCB1 diplotype (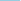) GCGC, (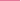) others, (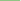) TTTT, (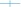) GCGC, (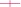) others, (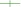) TTTT.

For the paclitaxel treatment group, patients with ABCB1 3435 TC and TT showed a higher risk of poor PFS when compared with individuals who had the 3435 CC genotype (TT 3.87 months vs. TC 9.50 months vs. CC 14.13 months；*P* < 0.001) (Fig 2a). Patients with ABCB1 2677 TG and TT genotype also indicated shorter PFS when compared with 2677 CC genotype (TT 4.37 months vs. TG 9.73 months vs. GG 12.1 months；*P* = 0.013) (Fig 2b). For haplotype analysis, patients with 2677T/A/3435T‐2677 T/A/3435T had the worst PFS (2677T/A/3435T‐2677 T/A/3435T 3.87 months vs. others 9.73 months vs. 2677G/3435C‐2677G/3435C 12.10 months; *P* = 0.033) (Fig 2c). Non‐small cell lung cancer patients with ABCB1 2677 and 3435 variants had a higher probability of shorter PFS for paclitaxel‐based chemotherapy.

Cox model analyses were performed individually within each of the three taxane treatment arms. Both the 2677 and 3435 polymorphisms, clinical characteristics (sex, age, smoking, pathology), dose adjustment, treatment line and combined platinum were included in the initial Cox model because of the different parameters worthy of exploration in the models. By backward elimination, we found that the 3435C > T polymorphism was an independent factor that was associated with poor PFS in the paclitaxel treatment group (HR of 7.70 for 3435 variants, 95% CI: 1.76–33.58, *P* = 0.023).

### Pharmacokinetic analysis

The AUC of 20 patients treated with docetaxel was analysed and the values of docetaxel showed substantial interpatient variability, with the AUC ranging from 1.2 to 4.8. The median docetaxel AUC was 3.2 mg*hour/L. No significant association was found between the pharmacokinetic parameters of docetaxel and age, gender, pathology, stage and smoking status, suggesting that the pharmacokinetic variability was independent of these factors. However, we found an increasing AUC with ABCB1 either 2677 or 3435 single nucleotide mutation. The difference between ABCB1 2677 locus was of statistical significance (*P* = 0.05, Table 6).

**Table 6:** Pharmacokinetic parameters of docetaxel

SNP	genotype	AUC (mg × hour/L)	*P*‐value
2677 G > T/A	GG	2.51 ± 0.96	
	GT	3.77 ± 0.59	0.05
	TT	3.10 ± 0.96	
3435 C > T	CC	2.43 ± 1.00	
	CT	3.26 ± 0.92	0.063
	TT	3.75 ± 0.31	

*P*‐value was calculated by *t*‐test.

## Discussion

ABCB1 encodes P‐glycoprotein, which enables the elimination and secretion of chemotherapeutic drugs.^10^ It has been suggested that variability in expression of P‐glycoprotein, caused by SNPs, may induce the interindividual differences in drug response and toxicity. ABCB1 gene variants have been studied in several types of solid tumors treated with chemotherapy including ovarian, gastric and breast cancer. However, the study on lung cancer is very limited and no reports have been seen from China. Therefore, the purpose of this study was to evaluate the association of ABCB1 polymorphisms, adverse event, PFS and pharmacokinetic parameters in Chinese lung cancer patients treated with taxane‐based chemotherapy.

In this study, the frequency of ABCB1 3435 T allele was 36.1%, which was similar to the frequency in the Asian population in a previous study^11^, ^12^ (*P* > 0.05)，but was significantly lower than in the European population^7^, ^9^, ^13^ (*P* < 0.05). This indicates the frequency of ABCB1 3435 C > T polymorphism has racial differences. The occurrence of SNP was higher in the European population compared with the Asian population. The frequency of ABCB1 2677 T/A allele in this study was 54.4%, which was similar to the occurrence frequency in Asian and European populations. Therefore, 2677 G > T/A does not have evident racial differences.

The data in our study suggest a possible genetic predisposition to the occurrence of a taxane‐induced adverse event regulated by ABCB1 polymorphism. For example, Kim *et al*.^14^ and Sissung *et al*.^15^ both found ABCB1 3435 T/T was significantly associated with docetaxel‐related neutropenia. Sissung *et al*.^7^ also found that patients with wild‐type ABCB1 3435C > T transition were less likely to develop clinically‐significant peripheral neuropathy. Likewise, our study also suggested that ABCB1 3435 TT/TC genotype were more likely to have neuritis (TT 88.2%, TC 22.2%, CC 21.6% *P* = 0.004) in taxane treatment. This indicates that ABCB1 genetic variants may be differentially expressed in the blood‐nerve barrier in a genotype‐dependent manner.

ABCB1 gene polymorphisms have been correlated with taxane response, even though the results have been inconsistent.^7^, ^16^ Previous studies,^9^, ^17^ indicated that in gastric cancer individuals with ABCB1 3435 CC had a longer PFS in taxane‐treated patients. In our study, lung cancer patients harboring ABCB1 2677 and 3435 site wild‐type had longer median PFS in the paclitaxel treatment group, which was consistent with the results in gastric cancer.^9^ In prostate cancer,^15^ patients carrying the 2677T‐3435T haplotype had a shorter median survival in the docetaxel treatment group (*P* = 0.045), which also confirmed our finding that ABCB1 2677 and 3435 site wild‐type is a positive prognostic factor. However, in ovarian cancer, ABCB1 2677 T or A alleles mutation patients had a better response to paclitaxel treatment.^6^ This study analyzed response rather than PFS, so might not be comparable to our study. Hoffmeyer *et al*. found that a synonymous polymorphism 3435C > T was correlated with the level of P‐gp expression.^5^ Polymorphisms of ABCB1 may increase the efflux of chemotherapeutic drugs from cancer cells and thus increase their elimination from the body, resulting in a decrease of plasma concentrations, thereby affecting their therapeutic efficacy.^18^

Our study also focused on the ABCB1 gene and its effect on pharmacokinetic markers for docetaxel toxicity. We found an increasing AUC with ABCB1 either 2677 or 3435 single nucleotide mutation. In our study, the ABCB1 mutation also indicated a higher occurrence of neurotoxicity. The higher occurrence of toxicity might occur due to a higher blood concentration. Extra *et al*. found AUC correlated with the percentage decrease of neutrophils in phase I and pharmacokinetic study of Taxotere (docetaxel).^19^ Bruno *et al*. proved that docetaxel clearance (CL) was a strong independent predictor (*P* < 0.0001) of both grade 4 neutropenia and febrile neutropenia.^20^ These results support our speculation.

There was limited retrospective data in our study. First, polymorphisms in ABCB1 2677 and 3435 site might decrease the expression of P‐gp, and thus reduce the efflux of drugs and increase blood concentration.^21^ However, the expression level and the transport capacity of P‐gp in each group were not measured in our study, therefore, it was not determined whether the function of P‐gp changed. Second, the sample size was too small, especially for patients with therapeutic drug monitoring (TDM). Large‐scale prospective studies should be carried out to demonstrate these results. Third, in our study some confounding factors existed, including pathology, previous treatment and taxane species.

In conclusion, the ABCB1 polymorphism G2677T/A and C3435T correlated with high AUC and poor taxane response in lung cancer and predict neurotoxicity of taxane‐based chemotherapy. ABCB1 mutation increases the efflux of taxanes through the cell membranes, thus increases the AUC in plasma and leads to a resistant, but highly toxic phenotype. Further study of ABCB1 polymorphism and its effect on P‐gp phenotype may help to uncover the underlying mechanisms.

## Disclosure

All grants were received from the government. All authors declare there is no conflict of interest.
